# Mesenchymal stromal cells: what have we learned so far about their therapeutic potential and mechanisms of action?

**DOI:** 10.1042/ETLS20210013

**Published:** 2021-09-08

**Authors:** Francesco Amadeo, Katherine Trivino Cepeda, James Littlewood, Bettina Wilm, Arthur Taylor, Patricia Murray

**Affiliations:** 1 Department of Molecular Physiology and Cell Signalling, Integrative Biology, University of Liverpool, Crown Street, L69 3GE Liverpool, U.K.; 2 Centre for Pre-clinical Imaging, Institute of Systems, Molecular and Integrative Biology, University of Liverpool, Crown Street, L69 3GE Liverpool, U.K.

**Keywords:** animal models, extracellular vesicles, mesenchymal stromal cells, MSC clinical trials, MSCs

## Abstract

Mesenchymal stromal cells (MSCs) have been found to be safe and effective in a wide range of animal models of human disease. MSCs have been tested in thousands of clinical trials, but results show that while these cells appear to be safe, they tend to lack efficacy. This has raised questions about whether animal models are useful for predicting efficacy in patients. However, a problem with animal studies is that there is a lack of standardisation in the models and MSC therapy regimes used; there appears to be publication bias towards studies reporting positive outcomes; and the reproducibility of results from animal experiments tends not to be confirmed prior to clinical translation. A further problem is that while some progress has been made towards investigating the mechanisms of action (MoA) of MSCs, we still fail to understand how they work. To make progress, it is important to ensure that prior to clinical translation, the beneficial effects of MSCs in animal studies are real and can be repeated by independent research groups. We also need to understand the MoA of MSCs to assess whether their effects are likely to be beneficial across different species. In this review, we give an overview of the current clinical picture of MSC therapies and discuss what we have learned from animal studies. We also give a comprehensive update of what we know about the MoA of MSCs, particularly in relation to their role in immunomodulation.

## Introduction

Mesenchymal stromal cells (MSCs) were first isolated from bone marrow (BM) in the 1960s by Friedenstein and colleagues, who reported an adherent, fibroblast-like, clonogenic non-hematopoietic cell population with a high replicative capacity *in vitro* [1,2]. Since then, MSCs have been isolated from many other sources, including adipose tissue [3], umbilical cord blood [4] and Wharton's Jelly [5]. Because of the range of tissues of origin and the different protocols and media used to purify them, three minimum common criteria have been suggested by the International Society of Cell and Gene Therapy for defining MSCs: plastic adherence; trilineage (adipogenic, chondrogenic, osteogenic) differentiation potential *in vitro*; expression of CD90, CD73 and CD105, together with the absence of haematopoietic markers such as CD45 [6]. Additional characteristics, such as low immunogenicity and high immunomodulatory capacity [7], make MSCs a promising cell therapy for suppressing inflammation and promoting tissue regeneration.

MSCs have already shown considerable therapeutic potential in animal models of various diseases, including kidney injury [8], cardiac disease [9] and a range of orthopaedic conditions [10]. However, clinical trials have been generally disappointing, either because they have failed to establish efficacy, or because the results have been inconclusive [11,12]. If MSCs are to fulfil their promise and improve patient health, it is important to understand why the promising results from animal studies are not translating to the clinic. Some of the potential reasons apply to all novel therapies (i.e. they are not specific to MSCs), and include the following: use of animal models that poorly mimic human disease; trials being undertaken before positive results have been shown to be reproducible in animal models; shortcomings in experimental design and/or reporting bias. Indeed, an analysis of novel therapies for neurological conditions found that of 160 interventions that had been described as positive in animal studies, only eight should have been considered for clinical translation, the others being insufficiently robust [13].

In addition, there are issues that are specific to MSCs, the most notable being that we do not fully understand the mechanisms whereby MSCs elicit their beneficial effects. If we lack knowledge of how MSCs work in animal models, then we cannot begin to understand why they fail to work consistently in the clinical setting. Safety and efficacy data are also essential for determining the risk:benefit ratio of MSC therapies so as to judge whether they would be appropriate for clinical use. To assess safety and efficacy, and to understand the mechanism of action (MoA) of MSCs, better knowledge of their *in vivo* biodistribution and fate is fundamental. By increasing our understanding of how MSCs behave *in vivo*, we will be able to develop more optimal treatment regimens, and will be better-placed to target MSC therapies to those patients who are most likely to benefit. The aim of this review is to outline the latest progress in MSC research and therapy, ranging from their current use in clinical trials, the advantages and limitations of preclinical models, and our current understanding of their MoA. The review is mainly focussed on the paracrine effects of MSCs, rather than on their ability to repair tissues by differentiating into specialised cell types. This is because it is becoming increasingly clear that the differentiation of administered MSCs rarely occurs *in vivo* and that their therapeutic effects are mediated by paracrine factors, representing a paradigm shift in the MSC field [14].

## The clinical picture

**Figure 1.**
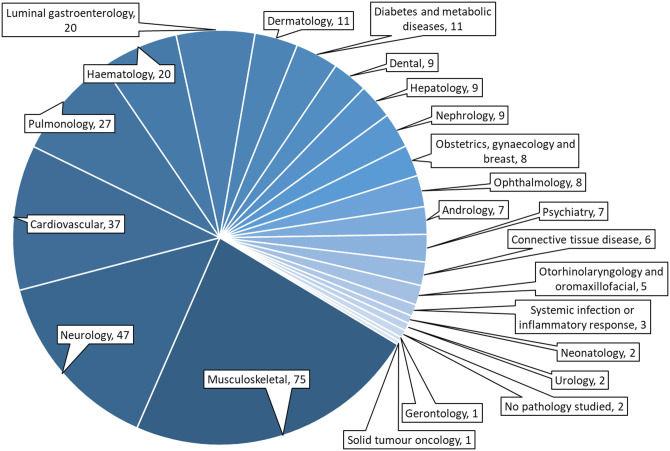
Distribution MSC trials registered as complete on clinicaltrials.gov, classified by specialty. A total of 327 studies were found using the terms ‘mesenchymal stem cells’ OR ‘mesenchymal stromal cells’.

At present, when searching on Clinical Trials.gov using the search terms ‘mesenchymal stem cells’ or ‘mesenchymal stromal cells’, there are over 3000 trials that are registered as completed. Of these, published results appear to be available for just 327, suggesting potential publication bias. The trials comprise a wide range of conditions, the most common being musculoskeletal (23%), neurological (14%) and cardiovascular (11%) (Figure 1). The majority of studies are uncontrolled and/or are Phase 1 trials that are limited to assessing safety, and fail to address efficacy. Although there have been some adverse outcomes, these have mostly occurred when patients have been administered MSC therapies from ‘for-profit’ cell therapy companies rather than through participating in a clinical trial [15]. Generally, when MSCs are administered via regulated and ethically approved clinical trials, they appear to be relatively safe [16]. However, whether they are efficacious or not is less clear. Efficacy can only be assessed in trials that include a control (i.e. placebo) group. Hence, in this review, we have limited our analysis to published studies that have been registered on Clinicaltrial.gov or on the European Union Drug Regulating Authorities Clinical Trials Database (EudraCT) and that include a control group (see Table 1). We have identified a number of common themes from these studies. Notably, only four studies included more than 100 patients, with the majority including only 10–30 patients. Hence, there is a need for larger patient cohorts to appropriately power for efficacy. Efficacy measures themselves differed significantly between studies, even when the same disease was being treated. Moreover, the link between efficacy measures to prognostic outcomes and their ability to show clinical significance for patients was not made. A variety of MSC sources, doses, administration routes, and number of treatment sessions were used in each category of disease and these different parameters were not usually assessed head-to-head. Another issue was that the MSCs were often used in conjunction with other interventions, such as biomaterial scaffolds, making it difficult to determine whether any observed efficacy was due to the MSCs, the scaffold, or the combination of the two. Some studies did appear to suggest evidence of efficacy [17,18], but where meta-analysis studies have been undertaken, they tend to show limited benefit [19,20]. In light of the generally disappointing outcomes, there is an argument for trying to understand more about the MoA of MSCs and to obtain reproducible efficacy from animal studies before undertaking more clinical trials.

**Table 1:** Characteristics of MSC trials registered on clinicaltrials.gov or EudraCT which included a control group and whose results have been published

Disease	MSC derived from	Autologous/allogeneic	Delivery route	References
Acute respiratory distress syndrome	Bone marrow	Allogeneic	Intravenous	[21]
Alveolar cleft	Adipose	Allogeneic	Intralesional	[22]
Angina	Adipose	Autologous	Intramyocardial	[23]
Autism	Umbilical cord	Allogeneic	Intravenous and intrathecal	[24]
Chronic obstructive pulmonary disease	Bone marrow	Allogeneic	Intravenous	[25]
Chronic obstructive pulmonary disease	Bone marrow	Allogeneic	Intralesional	[26]
Crohn's disease	Adipose	Allogeneic	Intralesional	[27]
Crohn's disease	Umbilical cord	Allogeneic	Intravenous	[28]
Crohn's disease	Adipose	Allogeneic	Intralesional	[29]
Degenerative disc disease	Bone marrow	Allogeneic	Intralesional	[30]
Degenerative disc disease	Not declared	Allogeneic	Intralesional	[31]
Diabetes foot ulcers	Adipose	Allogeneic	Topical	[32]
Diabetes mellitus	Bone marrow	Autologous	Intravenous	[33]
Diabetes mellitus	Bone marrow	Autologous	Intraarterial	[34]
Fracture	Adipose	Autologous	Intralesional	[35]
Fracture	Bone marrow	Autologous	Intralesional	[36]
Graft-versus-host disease	Bone marrow	Allogeneic	Intravenous	[37]
Heart failure	Umbilical cord	Allogeneic	Intravenous	[18]
Heart failure	Bone marrow	Autologous	Intramyocardial	[38]
Leukaemia	Umbilical cord	Allogeneic	Intravenous	[39]
Limb ischaemia	Bone marrow	Allogeneic	Intramuscular	[40]
Limb ischaemia	Bone marrow	Allogeneic	Intramuscular	[41]
Liver injury	Umbilical cord	Allogeneic	Intravenous	[42]
Liver injury	Bone marrow	Autologous	Intraarterial	[43]
Liver injury	Umbilical cord	Allogeneic	Intravenous	[44]
Motor neurone disease	Bone marrow	Autologous	Intrathecal and intramuscular	[45]
Multiple sclerosis	Adipose	Autologous	Intravenous	[46]
Multiple system atrophy	Bone marrow	Autologous	Intraarterial and intravenous	[47]
Myocardial infarction	Bone marrow	Autologous	Intraarterial	[48]
Myocardial infarction	Umbilical cord	Allogeneic	Intraarterial	[17]
Myocardial infarction	Bone marrow	Allogeneic	Intravenous	[49]
Myocardial infarction	Bone marrow	Allogeneic	Intravenous	[50]
Osteoarthritis	Adipose	Allogeneic	Intraarticular	[51]
Osteoarthritis	Adipose	Autologous	Intraarticular	[52]
Osteoarthritis	Umbilical cord	Allogeneic	Intraarticular	[53]
Osteoarthritis	Adipose	Autologous	Intraarticular	[54]
Osteoarthritis	Bone marrow	Autologous	Intraarticular	[55]
Osteoarthritis	Bone marrow	Autologous	Intraarticular	[56]
Osteoarthritis	Bone marrow	Allogeneic	Intraarticular	[57]
Osteoarthritis	Bone marrow	Autologous	Intraarticular	[58]
Parry-Romberg disease	Adipose	Autologous	Intralesional	[59]
Pulmonary fibrosis	Bone marrow	Allogeneic	Intravenous	[60]
Renovascular disease	Adipose	Autologous	Intraarterial	[61]
Rheumatoid arthritis	Bone marrow	Autologous	Intraarticular	[62]
Rheumatoid arthritis	Adipose	Allogeneic	Intravenous	[63]
Scar	Adipose	Not declared	Intralesional	[64]
Solid organ transplant	Bone marrow	Autologous	Intravenous	[65]
Spinal cord injury	Bone marrow	Autologous	Intrathecal	[66]
Spinal cord injury	Umbilical cord	Allogeneic	Intralesional	[67]

## What information can we obtain from animal models?

One of the aims of preclinical studies involving animals is to provide evidence of safety and efficacy of cell therapy products prior to them being applied in patients. Animal studies can also be very useful for establishing the optimal dose; the number of doses; the optimal route and timing of cell administration; and the optimal cell source (for instance, in the case of MSCs, whether bone marrow, adipose or umbilical cord-derived MSCs are most suitable for the specific condition being treated). However, this sort of systematic approach is seldom, if ever, undertaken. More typically, MSC therapies tend to be translated to the clinic before these important parameters have been fully established, and prior to the same treatment regime having been shown to give similar outcomes when used by two or more independent research groups. Consequently, as indicated in a recent review of stem cell therapies for heart disease, there is an urgent need for standardisation in preclinical studies [68]. Table 2 shows a collection of preclinical studies assessing MSC therapies for cardiac, lung and kidney disease, where it can be seen that no two studies are the same. Generally, most publications assessing the potential of MSC therapies in animal models tend to report statistically significant beneficial effects. This leads to concerns that animal models may not be good predictors of how MSCs will behave in human patients, given that most clinical studies have been disappointing. However, it is now recognised that due to publication bias, negative results are less likely to be reported [69], a recent evaluation of two German university medical centres indicating that only 58% of animal studies were published in research articles [70]. A more extensive analysis by van der Naald et al. [71] found that while ∼60% of animal studies were published, outcomes for only 26% of the animals used in the studies were reported. It is important to improve this situation because negative outcomes are just as important as positive ones, and give crucial information about whether a particular MSC therapy may be likely to be beneficial in the clinic. One way to address this problem would be to require preregistration of all animal studies [71], similarly to how clinical trials are now pre-registered on databases such as Clinical Trials.gov.

**Table 2:** Selection of preclinical studies assessing MSC therapies for cardiac, lung and kidney disease, where it can be seen that no two studies are the same

Organ	Model	Number of animals	Cell source	Dose (numbers of cells transplanted)	Administration route	Time point of administration	Length of follow-up after therapy	References
Heart	Myocardial-infarction by occlusion-reperfusion in pig model	*n* = 22 — 2* died during ‘peri’ procedure	Xenogeneic human BM-derived cells of chronic heart failure patients	5 × 10^7^ cells in 300 µl	Intra- myocardial (delivered into the infarction border-zone)	30 days after MI induction	30 days	[72]
	Acute myocardial infraction by coronary occlusion in sheep	44 sheep — 3* died during procedure — 3* after injection	Allogenic MSCs overexpressing mutant human hypoxia-inducible factor 1α	2 × 10^7^ cells in 2 ml PBS	Intramyocardially injected in the peri-infarct zone (10 aliquots of 20 µl)	30 min after ligation of left anterior descending coronary artery	1, 30, 60 days	[73]
	Acute myocardial infarction in mini pigs	20 (15 survived)	Allogeneic, male BM-MSCs	50 million MSCs in 9ml PBS	Intracoronary transplantation + three boluses	6–8 days after myocardial	15 days	[74]
	Acute myocardial infarction in rat model	110 — *27 died after procedure	Allogeneic BM — MSCs from 3 — week — old male Lewis rats	1 × 10^6^ MSCs in <25 ml saline or PBS	Intramyocardially injection (peri-infarcted area/one site per heart)	2 weeks after myocardial infarction	3, 7, 14, 18 days	[75]
	Murine IRI model	17	Allogeneic mouse AD-MSCs	3.5 × 10^5^ cells 15 µl saline	Trans-epicardial	10 min after reperfusion	1, 3, 7 days	[76]
Lung	Acute lung injury in mice	64	human UC-MSCs	1 × 10^6^ cells in 200 µl saline	Tail vein injection	4 h after injury	30 min, 1, 3, 7 days	[77]
	Pulmonary fibrosis in mice	49	38 to 40- week healthy term human UC-MSCs	5 × 10^5^ cell/mouse in 50uL sterile PBS	Intra-tracheally	15 min after bleomycin instillation	21 days	[78]
	Acute respiratory distress syndrome	10	AD-MSCs	200 × 10^7^ cells	Intravenously over 30 min via central line	1 h after injury	24, 48 h	[79]
Kidneys	IRI in rats	24	human UC-MSCs	1 × 10^6^ cells/rat	Tail vein	Unknown	30 days	[80]
	Cisplatin-induced acute kidney injury in mice	70	Allogenic mouse AD-MSCs	2.5 × 10^7^ cells/kg	Intravenous infusion	Unknown	7 days	[81]
	Cisplatin-induced acute kidney injury in rats	20	Human kidney-derived cells expressing CD133	1 × 10^6^ cells/500 ml PBS + second dose 7 days later	Tail vein injection	2 days after cisplatin	2, 7, 14 days	[82]
	Renal IRI in rats	18	Allogeneic BM-MSCs	2 × 10^6^ cells	Injected into the renal artery	one week after IRI	1, 7, 14, 21 days	[83]

## What do we know about the therapeutic mechanisms of MSCs?

**Figure 2.**
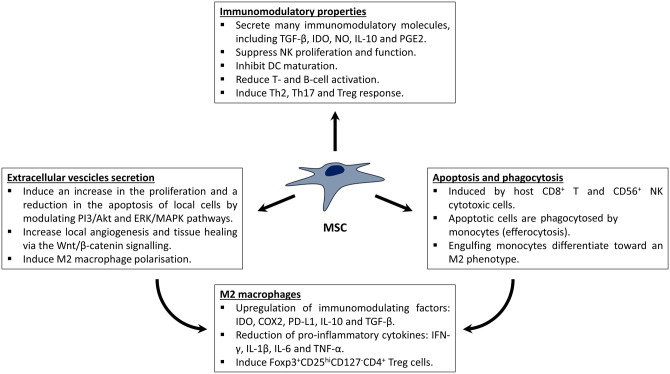
Potential mechanisms by which MSCs might act.

Accumulating evidence suggests that MSCs can exert their therapeutic potential by modulating the immune system instead of by replacing damaged cells and tissues (Figure 2). Different *in vitro* and *in vivo* studies have shown that MSCs can regulate both the innate and adaptive immune systems by suppressing natural killer cell proliferation and function, inhibiting dendritic cell maturation, reducing B and T cell activation and by increasing the differentiation of T cells toward a regulatory phenotype [84]. MSCs secrete many soluble factors capable of mediating their immunomodulatory effects, including (i) transforming growth factor-β1 (TGF-β1), involved in the regulation of lymphocyte proliferation, differentiation and survival; (ii) indoleamine-pyrrole 2,3-dioxygenase (IDO), an enzyme involved in the degradation of tryptophan, required for T cell activity; (iii) nitric oxide (NO), which attenuates T cell responsiveness; (iv) interleukin-10 (IL-10), a potent anti-inflammatory cytokine; and (v) prostaglandin E2 (PGE2), which suppresses the effector functions of macrophages, neutrophils and dendritic cells, but promotes Th2, Th17, and Treg responses [84–86] (Figure 2).

In most preclinical studies involving small animals, MSCs are administered intravenously (IV) [87,88], which leads to them being entrapped in the microcapillary network of the lungs, where most of the cells die within 24–48 h [87,89–91]. Therefore, one of the main questions about MSCs is: how can these cells exert their therapeutic function if after IV injection, they get sequestered in the lungs and disappear after a few days? The mechanisms responsible for the clearance of infused cells in the lungs are not yet fully elucidated.

Recognition and engulfment of apoptotic cells by phagocytic cells have an important role in tissue homeostasis, immunomodulation and the regulation of inflammation. When a cell undergoes apoptosis, it is cleared by local macrophages, which can then polarise towards different phenotypes depending on the stimulus. Apoptosis and phagocytosis have been recently proposed as mechanisms involved in immunomodulation mediated by MSCs after IV injection and lung entrapment [85,90,92,93] (Figure 2). Dazzi and co-workers used a mouse model of graft versus host disease (GVHD) to investigate the role of MSC apoptosis and phagocytosis in immunomodulation [85]. Intravenously infused bone marrow MSCs (BM-MSCs) rapidly underwent extensive caspase activation and apoptosis, without affecting their immunosuppressive function. This apoptotic effect was mediated by the release of granzyme B and perforin by host CD56^+^ Natural Killer and CD8^+^ T cytotoxic cells. Interestingly, the recognition of MSCs was not triggered nor mediated by the human leukocyte antigen (HLA) class I or class II, and the formation of an immunological synapse was not required. Dazzi and co-workers confirmed the role of MSC apoptosis in mediating immunomodulation by infusing apoptotic MSCs (apoMSCs) and obtaining the same immunomodulatory effect [85]. A subsequent study by the same group showed that culturing monocytes with apoMSCs can lead to a reduction in the T-cell response [94]. Interestingly, these monocytes exhibited a functional and molecular immunosuppressive phenotype, with significant up-regulation of immunomodulatory molecules, including IDO, cyclooxygenase2 (COX2) and programmed death-ligand 1 (PD-L1), together with an increased secretion of PGE2 and IL-10, and a reduction in tumour necrosis factor-α (TNF-α) [94]. The up-regulation of PD-L1, IDO and IL-10 by the COX2/PGE2 axis was also demonstrated, identifying it as a key effector of apoMSC-induced immunosuppression. Indeed, only monocytes that have engulfed the apoMSCs displayed this phenotype, linking the *in vivo* MSC apoptosis with their immunomodulatory function [94]. This result agrees with a recent study published by de Witte and colleagues [90], where human umbilical cord MSCs (UC-MSCs) labelled with the lipophilic dye PKH26 were infused in mice. As expected, the cells were trapped in the lungs and after 24 h, the PKH26 dye was mostly found in CD11+ cells, suggesting that the UC-MSCs had been phagocytosed by the host's innate immune cells. de Witte and colleagues confirmed these results *in vitro*, showing MSCs could shift macrophages from a pro-inflammatory phenotype to an intermediate one, which in turn, up-regulated the level of Foxp3^+^ CD25^hi^ CD127^low^ CD4^+^ regulatory T cells (Tregs) [90].

A similar mechanism was reported by Braza et al. [93], who found that IV infused PKH26-labelled BM-MSCs were cleared in the lungs by monocytes/macrophages within 24 h. The PKH26 positive macrophages displayed a M2 phenotype, and secreted higher levels of TGF-β and IL-10. A more recent study has shown that macrophages adopt a regulatory-like phenotype after the efferocytosis of adipose MSCs (AD-MSCs); this was accompanied by an up-regulation of IL-10 secretion, and a reduction in TNF-α and NO [95]. A possible mechanism to explain the phagocytosis mediated by macrophages was proposed by Gavin et al. [92], who found that live MSCs can be phagocytised by monocytes via a complement-mediated opsonisation. The complement system is made up of a large spectrum of different plasma proteins that can react with each other to opsonise pathogens and trigger a series of inflammatory responses. After exposing BM-MSCs to human plasma, an enrichment of C3 complement protein was detected on the surface of the cells [92]. Interestingly, an increase in monocyte phagocytosis was observed when MSCs were pre-treated with plasma, but this effect was significantly reduced following inhibition of the C3 protein [92], suggesting a direct role of complement opsonisation in the clearance of the infused cells.

Taken together, these results suggest a direct involvement of the immune system in the clearance of infused MSCs and in mediating their function. After MSCs get trapped in the lungs, they are quickly sacrificed and opsonised by local cytotoxic cells and macrophages, respectively. Then, the phagocytosis triggers the polarisation of the macrophages to a M2 immunomodulatory phenotype, which can increase the secretion of immunomodulatory factors, such as IDO, IL-10 and TGF-β, and activate Treg cells. Nevertheless, even if the involvement of phagocytosis and MSC clearance after IV infusion is becoming clearer, how this mechanism can ameliorate tissue damage in the host remains to be elucidated.

## MSC-derived extracellular vesicles

Another potential mechanism for the therapeutic effect of MSCs is via the release of extracellular vesicles (EVs) (Figure 2). These membrane-bound vesicles contain proteins, nucleic acids and lipids, some of which could potentially mediate the effects of MSCs. Indeed, during the last few years, EVs derived from different sources of MSCs were found to have a therapeutic effect in many disease models, such as myocardial ischemia-reperfusion injury (IRI) [96,97], renal IRI [98], wound healing [99], hepatic disease [100,101], cartilage and bone regeneration [102–104] and neurological disease [105]. In particular, many studies have reported an increase in local cell proliferation and reduction in apoptosis and inflammation after EV infusion or transplantation [106,107], and different molecular mechanism have been investigated. A molecular pathway that has recently been found to be regulated by MSC-derived EVs and to have a role in regulating proliferation and apoptosis, is the protein kinase B (also known as Akt), extracellular receptor kinase (ERK) and mitogen-activated protein kinase (MAPK) axis [103,104,107,108]. The phosphatidylinositol 3-kinase (PI3K)/Akt pathway and MAPK/ERK signalling cascade both comprise a group of downstream effectors important for regulating cell proliferation, survival and apoptosis, and invasion [109,110]. In a recent study involving both an *in vitro* and *in vivo* model of osteoarthritis (OA), Zhang et al. [104] demonstrated that MSC-derived EVs could reduce inflammation and restore matrix homeostasis by acting through adenosine receptor-mediated Akt and MAPK/ERK phosphorylation on local chondrocytes; this led to an increase in local proliferation, and a reduction in apoptosis and fibrosis [104]. A previous report by the same group showed that the activation of these two pathways in chondrocytes was mediated by the ecto-5′-nucleotidase (NT5E) activity of CD73 [107], an enzyme that is enriched in EVs derived from certain cell types. CD73 is able to convert extracellular adenosine monophosphate to adenosine, which in turn can interact with adenosine receptors and modulate the Akt and MAPK/ERK signalling pathways [111,112]. These results were confirmed by Chew and colleagues, who demonstrated the involvement of this mechanism in the enhancement of periodontal regeneration mediated by MSC-derived EVs [103].

The Wnt/β-catenin signalling pathway, which plays a key role in tissue homeostasis and cell fate [113], may also play a role in EV-mediated tissue regeneration, as evidenced by the involvement of this pathway in wound healing following exposure to MSC-derived EVs [114,115] . The subcutaneous injection of human UC–MSC derived EVs in a rat wound injury model increased local angiogenesis and healing, but this effect was reduced following Wnt4 knockdown [114]. The involvement of Wnt/β-catenin was also revealed in a model of myocardial IRI, where increased activation of the pathway in the rat myocardium following AD-MSC derived EV administration, was associated with an increased survival of local cardiomyocytes [97]. However, a study reporting that BM-MSC derived EVs can reduce liver fibrosis suggests that this effect may result from inhibition of the Wnt/β-catenin pathway [116]. Further analysis is therefore required to clarify the effect of MSC-derived EVs on the Wnt/β-catenin pathway, and to establish its significance in tissue repair.

EVs can also play a role in immunomodulation, a recent metabolomic study indicating how priming MSCs through exposure to specific culture conditions, can increase the packaging of immunomodulatory molecules and lipid membrane components inside the EVs [117]. As a direct consequence, administration of MSC-derived EVs resulted in an increase in M2 macrophage infiltration and anti-inflammatory cytokine up-regulation, with a parallel decrease in M1 macrophages and pro-inflammatory cytokines [96,107,118]. Evidence suggests that the effect of MSC-derived EVs on macrophage polarisation is crucial for MSC-mediated wound healing. Indeed, the depletion of macrophages can reduce and delay MSC-induced wound healing [96,119], and the same effect can be obtained by inhibiting the release of EVs, which also results in a reduction in M2 polarisation [119].

The polarisation of macrophages towards an M2 phenotype after exposure to MSC-derived EVs is becoming quite well established [96,107,120–122] and different factors have recently been proposed to be involved in this. Lan and co-workers showed how the incorporation of protein inside the EVs can induce effects that the free form of the same protein could not do; for instance, they discovered that the incorporation the immune regulator, Metallothionein-2, into EVs, could increase the activity of anti-inflammatory macrophages, whereas the free form of this protein has no effect [118]. Apart from proteins, EVs also contain miRNAs, many of which can have immunomodulatory effects. For instance, Let-7a, miR-23a, miR-25b [122] and miR-182 [96] have already been shown to down-regulate the Toll-like receptor 4 (TLR4)/NF-kB signalling pathway within macrophages, which in turn, increases the activation of the PI3K/Akt signalling pathway, leading to M2 macrophage polarisation [96].

Even if there is no clarification yet whether there is a polarisation of the local macrophages or just a recruitment of these cells, all these results support the active role of immunomodulatory macrophages in mediating any potential therapeutic effects of MSCs, and the involvement of MSC-secreted EVs in this mechanism. However, the *in vivo* biodistribution, pharmacokinetics and the specific mechanism of action of exogenously administered EVs have yet to be elucidated.

## Summary

- MSCs have shown efficacy in a wide range of animal models of human disease, but lack of standardisation in how the therapies are developed and administered, means there are concerns regarding reproducibility. There is little evidence that animal studies are repeated by independent research groups to confirm safety and efficacy of MSC therapies before progression to clinical trials.
- Thousands of clinical trials have been conducted that have assessed the potential of MSC therapies in a variety of conditions. Generally, while MSCs appear to be safe, most trials show limited, if any, efficacy.
- Before undertaking more clinical trials, in addition to confirming reproducibility in animal studies, it is important to understand the MoA of MSCs more fully. Recent studies indicate that the therapeutic effects of MSCs are mediated by paracrine factors, including MSC-derived EVs, and that in some cases at least, appear to promote repair by modulating the host's immune system. A greater understanding of the MoA of MSCs will hopefully allow MSC-based therapies to be better targeted in the future.

## Open Access

Open access for this article was enabled by the participation of University of Liverpool in an all-inclusive *Read & Publish* pilot with Portland Press and the Biochemical Society under a transformative agreement with JISC.

## Abbreviations

AD-MSCs: adipose MSCs
BM: bone marrow
BM-MSCs: bone marrow MSCs
COX2: cyclooxygenase2
ERK: extracellular receptor kinase
EudraCT: the European Union Drug Regulating Authorities Clinical Trials Database
EVs: extracellular vesicles
GVHD: graft versus host disease
IDO: indoleamine-pyrrole 2,3-dioxygenase
IL-10: interleukin-10
IRI: ischemia-reperfusion injury
MAPK: mitogen-activated protein kinase
MoA: mechanism of action
MSC: mesenchymal stromal cell
NO: nitric oxide
NT5E: ecto-5′-nucleotidase
PD-L1: programmed death-ligand 1
PGE2: prostaglandin E2
PI3K: phosphatidylinositol 3-kinase
TGF-β1: transforming growth factor- β1
TLR4: Toll-like receptor 4
TNF-α: tumour necrosis factor-α
Tregs: regulatory T cells
UC-MSCs: umbilical cord MSCs
